# Effect of the clinical application of the diode laser (810 nm) in the treatment of dentine hypersensitivity

**DOI:** 10.1186/1756-0500-7-31

**Published:** 2014-01-13

**Authors:** Nada Tawfig Hashim, Bakri Gobara Gasmalla, Ahmed Hassan Sabahelkheir, Alhadi Mohieldin Awooda

**Affiliations:** 1 Khartoum North Dental Hospital, Faculty of Dentistry University of Sciences and Technology, Khartoum, Sudan; 2 Department of Oral Rehabilitation, Faculty of Dentistry, University of Khartoum, Khartoum, Sudan; 3 Laser Institute, Sudan University for Sciences and Technology, Khartoum, Sudan; 4 Department of restorative dentistry, University of Medical Sciences and Technology, Khartoum, Sudan

**Keywords:** Dentine sensitivity, Laser therapy

## Abstract

**Background:**

Dentine hypersensitivity is a common clinical finding with a wide variation in prevalence values. The aim of this study was to evaluate the use of diode laser (810 nm) in the treatment of cervical dentine hypersensitivity.

**Methods:**

Five patients, with at least two sensitive teeth were selected. A total of 14 teeth were included in this trial. By using Visual Analogous Scale the pain of dentine hyper sensitivity was detected and the pre- treatment readings were recorded.

The Diode laser (810 nm), was irradiated on (non contact) mode at the cervical region.

The samples were divided into two groups according to exposure duration: For Group 1 exposure duration was 30 seconds and for group 2 exposure duration was one minute.

The efficiency of the treatment was assessed at two examination period :15 minutes after first application and 7 days after first application, the degree of sensitivity was determined by using Visual Analogous Scale.

**Results:**

The results show significant reduction of pain after 15 minutes of laser application in the group with 30 seconds exposure duration (P = .001), and the pain completely fade away after one week in the same group, while in the group with 1 minute exposure duration the pain completely disappeared (visual analogous scale = (0)) after 15 minutes and one week of laser application (P = 0.001).

**Conclusion:**

The study concluded that application of diode laser (810 nm) was effective for the reduction of dentine hypersensitivity.

## Background

Dentine hypersensitivity is prevalent amongst a large population of individuals with 30 to 40 years of age. Malnutrition due to dietary restrictions in some individuals causes oral discomfort and pain in some individuals. The nociceptive stimulus commonly reported in the majority of cases is that of cold, followed by the mechanical stimulus of tooth brushing and the chemical stimulus of diet with a high concentration of sugar [1].

Pain of dentine hypersensitivity is sharp, localized and of short duration. The hydrodynamic theory proposed by Brännströn Aström in 1964 [2] is the most acceptable theory in explaining the relationship between the pain of dentine hypersensitivity and the displacement of biological fluid in the dentinal tubules. Thermal, physical and chemical stimuli may cause the displacement of the pulp-dentine fluid, which trigger pulpal nervous terminations.

Under normal conditions, dentine is covered by enamel or cement and does not suffer direct stimulation. Only with the exposure of peripheral terminations of dentinal tubules a situation of strong dentinal sensitivity manifests itself as “hypersensitivity”. Occlusion of the exposed dentinal tubules can reduce the intensity of dentinal sensitivity. The most common clinical cause for exposed dentinal tubules is gingival recession. A wide range of mechanisms has been proposed as causes of recession such as abrasion due to faulty tooth brushing; abfraction; parafunctional habits or occlusal disequilibrium. Erosion has also been implicated in gingival recession, with its deleterious effect of acids in the oral cavity; as well as improperly controlled dentinal acid conditioning [1].

When gingival recession occurs, cementum is exposed. Cementum, is easily abraded or eroded away which exposing the underlying dentine [3].

Treatment of dentine hypersensitivity is challenging for both the patient and the healthcare provider and this can be due to the following reasons: It is difficult to measure/compare pain in different individuals and patients hardly change their habits that initially caused the problem. Hypersensitivity may be resolved without any treatment or may require several weeks of applying desensitizing agents before improvement is seen [4].

There are two principal treatments either to desensitize the nerve with potassium nitrate or to cover the dentinal tubules with composite restoration, glass ionemer cement, periodontal graft and crown placement [3].

With the advent of laser into the field of clinical dentistry a hope of overcoming some of the drawbacks of conventional dental procedures was raised. Since its first experiment for dental application in 1960s, the use of laser has been increased rapidly in the last couple of decades. Nowadays, wide varieties of procedures are carried out using lasers. Lasers are found to be effective in cavity preparation, caries removal, restoration removal, etching, and treatment of dentine sensitivity, caries prevention and bleaching [5] which are accomplished by the interaction of laser with the tissue, causing different tissue reactions, according to its active medium, wavelength and power density and to the optical properties of the target tissue [6].

Dentine Hypersensitivity is a challenging problem in dentistry with an increasing of its prevalence nowadays. Due to the availability of laser treatment and its ease of application this study was carried out among Sudanese patients with cervical dentine sensitivity for the first time.

The aim of this study is to assess the effect of diode laser (810 nm), with different exposure duration, in the treatment of dentin hypersensitivity.

## Methods

A total of 14 teeth of 5 individuals (2 males and 3 females; aged 25 to 35 years old) with cervical dentine hypersensitivity were treated. Approval by the Ethics Committee and informed written consent was obtained at the clinic of Laser Institute, Sudan University of Science and Technology. The teeth included in the research were caries –free and showed no signs of active periodontal disease. The individuals with dentine hypersensitivity were recommended not to take any analgesics, anticonvulsants antihistaminic, sedative, tranquilizing or anti-inflammatory medications in the 72 hours preceding laser treatment, and not to use any desensitizer dentifrices in the last 6 weeks, and they should not been subjected to periodontal surgery in the last 6 months.

The tactile test was performed by the contact to the cervical dentine surface with a periodontal probe.

The samples were divided into 2 groups of 7 teeth: the 30 seconds exposure duration group and the 60 seconds exposure duration group.

The applied laser device was Diode Laser (810 nm) Oralia Company – Germany, with power of 20 Watt, class III (b).

This study was performed by one operator and one assessor responsible for the measurement of the pain level of the patients. The treatments were carried out in 2 sessions, a weekly interval.

The samples were evaluated by measuring the response of dentine hypersensitivity to the tactile stimulus. Scores were recorded in an analogous visual scale: score 10 (unbearable pain); 7 to 9 (strong and bearable pain), 4 to 6 (moderate pain), 1 to 3 (light pain) and 0 (no pain) [7]. The measurements were performed before each treatment session and at 15 min after laser application. This result was called immediate effect. Additional measurement was also performed at seven days after the laser application. This result was called late effect.

Except the exposure duration the irradiation parameters were identical, one of 30 seconds and other of 60 seconds (1 min). The power was one watt continuous emission form, and application with non contact mode on the region of exposed dentine.

Regarding the statistical analysis, all of the studied variables were described in relation to the mean and standard deviation. In order to compare the studied variables, Freidman test and Chi square test were applied; Two-Sample Kolmogorov-Smirnov Test was used for comparison of the two groups (30 seconds- 1 minute) after 15 minutes and after 1 week. The significance level of the test was .05.

## Results

**Figure 1:**
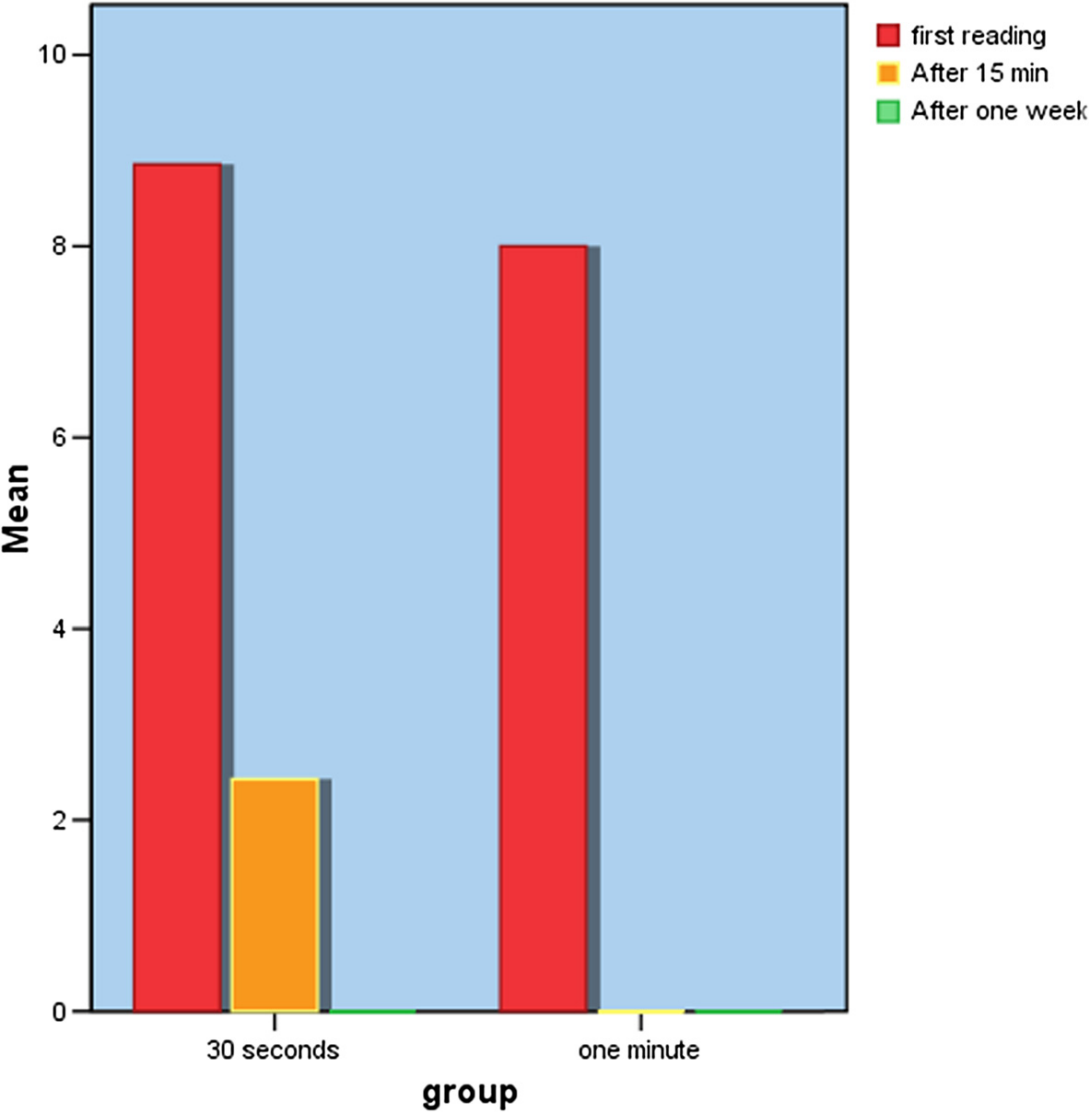
Comparison between the reading of pain before laser application, after 15 minutes from the application and after one week in both groups.

Significant reduction of dentine sensitivity occurred along all times measured during the two treatment sessions in both groups treated with 30 seconds exposure duration and 60 seconds exposure duration. Comparing the responses in the 2 treatment sessions of the 2 groups, by using Kolmogorov- Smirnov test as shown in (Table 1) there was statistically significant difference ( p = .002) between the two groups after 15 minutes of laser application , z = 1.871. There is no difference between the two groups after one week, P = 1.000 (Figure 1).

**Table 1:** Comparison between the two groups (30 seconds- 1 minute) after 15 minutes and after 1 week

	**After 15 minutes**	**After 1 week**
**Kolmogorov- Smirnov Z**	**1.871**	**.000**
**Significance**	**.002**	**1.000**

The results of treatments with the 30 seconds and 60 seconds exposure durations are reported in Tables 2 and 3 and statistical results are reported in Tables 4 and 5.

**Table 2:** Group 1 with 30 seconds exposure duration

	**N**	**Mean**	**Std. deviation**	**Minimum reading**	**Maximum reading**
**First reading**	**7**	**8.86**	**.378**	**8**	**9**
**After 15 minute**	**7**	**2.43**	**.535**	**2**	**3**
**After one week**	**7**	**.00**	**.000**	**0**	**0**

**Table 3:** Group 2 with 1 minute exposure duration

	**N**	**Mean**	**Std. deviation**	**Minimum**	**Maximum**
**First reading**	**7**	**8.00**	**1.528**	**5**	**9**
**After 15 minutes**	**7**	**.00**	**.000**	**0**	**0**
**After one week**	**7**	**.00**	**.000**	**0**	**0**

**Table 4:** Friedman test, group (1) 30 seconds

**N**	**7**
**Chi-Square**	**14.000**
**df**	**2**
**Significance**	**.001**

**Table 5:** Friedman Test, group (2) with 1 minute exposure duration

**N**	**7**
**Chi- Square**	**14.000**
**df**	**2**
**Significance**	**.001**

## Discussion

Although several types of treatments for dentine hypersensitivity have been demonstrated in the literature , there is no treatment that reduces pain to satisfactory levels.

Pain resulting from dentine hypersensitivity can be eliminated by interruption of stimuli transmission to the nerve endings of odontoblast processes which can be accomplished by reducing the fluid movement inside the dentinal canalicules, by narrowing or occlusion of tubules openings [8]. However, other treatment modalities have been proposed, such as laser therapy.

The use of He–Ne and GaAlAs lasers has shown a reduction in sensitivity to thermal and tactile stimuli [9,10]. Accordingly, in the present study, laser therapy promoted a considerable decrease in sensitivity, after 15 minutes and 7 days of the first application.

In this conducted research, it was observed that teeth, which presented exacerbated sharp pain during air blasting and tactile touch with dental instrument and continuous discomfort after the removal of these stimulation before desensitizing treatments were accomplished, showed an accentuated decrease of painful sensation immediately after 15 minutes of the first application of diode laser (810 nm) and even one week after initial irradiation. The rapid desensitizing effect of laser therapy observed in the conducted research may be attributed to a mechanism through which diode laser can induce changes in neural transmission networks within the pulp (depressed nerve transmission), rather than alterations in the exposed dentine surface, as observed with other treatment modalities [11]. In addition laser therapy may stimulate the normal physiological cellular functions. Therefore, the laser would stimulate the production of sclerotic dentine, thus promoting the internal obliteration of dentinal tubules [12] and this suggestion is reinforced by the histological analyses of dental pulps in teeth carried out by Matsumoto [13] the study showed a better degree of repair at 14 and 30 days after laser irradiation when compared with the non-irradiated dental pulp group.

The laser irradiation contributed to the repair of the dentine-pulp complex, preserving the pulpal vitality.

## Conclusions

Based on the results obtained in the treatment of 14 teeth with cervical dentine hypersensitivity, by measuring the initial response between grade 8-9 in the quantitative numeric pain scale (maximum of the pain scale), we conclude that: 1) Diode laser (810 nm) provided a decrease in cervical dentine hypersensitivity.

2) The therapeutic immediate and late effects of the diode laser 810 nm with 60 seconds exposure duration were greater than those of the 810 nm with 30 seconds exposure duration.

## Abbreviations

GaAlAs: Gallium,. aluminum and arsenide; He-Ne laser: Helium and neon.

## Competing interests

The authors declare that they have no competing interests.

## Authors’ contributions

NTH designed the study and carried out the data collection and the data analysis, AMA and AHS supervised the research, NTH writing/editing of the article with assistant of BGG. All authors have read and approved the final manuscript.
